# Transcriptome profiling for floral development in reblooming cultivar ‘High Noon’ of *Paeonia suffruticosa*

**DOI:** 10.1038/s41597-019-0240-1

**Published:** 2019-10-22

**Authors:** Yanting Chang, Tao Hu, Wenbo Zhang, Lin Zhou, Yan Wang, Zehui Jiang

**Affiliations:** 1 Key Laboratory of Bamboo and Rattan Science and Technology, State Forestry Administration, Institute of Horticultural Flower and Landscape, International Center for Bamboo and Rattan, Futongdong Rd, Wang Jing, Chaoyang District Beijing 100102 China; 2 State Key Laboratory of Tree Genetics and Breeding, Key Laboratory of Tree Breeding and Cultivation of State Forestry Administration, Research Institute of Forestry, Chinese Academy of Forestry, Beijing, 100091 China

**Keywords:** Shoot apical meristem, Development, Transcriptomics

## Abstract

Tree peony (*Paeonia suffruticosa* Andrew) is a popular ornamental plant due to its large, fragrant and colorful flowers. The floral development is the most important event in its lifecycle. To explore the mechanism that regulate flower development, we sequenced the flower bud transcriptomes of ‘High Noon’, a reblooming cultivar of *P*. *suffruticosa* × *P*. *lutea*, using both full-length isoform-sequencing (ISO-seq) and RNA-seq were sequenced. A total of 15.94 Gb raw data were generated in full-length transcriptome sequencing of the 3 floral developmental stages, resulting 0.11 M protein-coding transcripts. Over 457.0 million reads were obtained by RNA-seq in the 3 floral buds. Here, we openly released the full-length transcriptome database of ‘High Noon’ and RNA-seq database of floral development. These databases can provide a fundamental genetic information of tree peony to investigate its transcript structure, variants and evolution. Data will facilitate to deep analyses of the transcriptome for flower development.

## Abstract (summary)

**Table:** 

Measurement(s)	transcription profiling assay • full-length isoform
Technology Type(s)	RNA sequencing • isoform sequencing
Factor Type(s)	developmental stage
Sample Characteristic - Organism	Paeonia suffruticosa

Machine-accessible metadata file describing the reported data: 10.6084/m9.figshare.9938360

## Background & Summary

Tree peony (*Paeonia suffruticosa* Andrew), is one of the most important horticultural plants in the world and a culturally important ornamental plants in China, due to its striking ornamental and medicinal values. It is a perennial deciduous shrub with large, fragrant, and colorful flowers. With a long history of cultivation, there are more than 3,000 cultivars all over the world. ‘High Noon’ (*P*. *suffruticosa* × *P*. *lutea*) is one of the most famous and popular cultivars and is always used for hybrid breeding due to its characteristic of cup shape, semi-double and clear lemon color. On the other hand, High Noon showed an unregular reblooming phenomenon^1^ which means a twice floral development occurred around a year. These traits made ‘High Noon’ a suitable material for researching the floral development in tree peony.

Floral development is the most important developmental event in the life cycle of higher plants. The flowering timing is determined by processes of flowering transition, floral bud differentiation and floral organ identification^2^. A complex gene regulatory network was involved in the floral bud differentiation including plant hormone signal pathway and meristem activity regulation. Although there are great progresses in the study of molecular mechanism in floral development of model plants, it remains unclear in perennial plants, especially tree peony, whose genome information was not yet published. A few genes have been identified to be involved in the transition of shoot apical meristem (SAM) to floral bud in tree peony, including *SOC1*^3^, *FT*^4^, and *AP1*^5^. However, it was still hard to understand the underlying mechanism on floral development of tree peony at transcriptome level.

Next-generation sequencing (NGS) provides precise and comprehensive analysis of RNA transcripts for gene expression. It is applied to explore biological research frequently. Single molecular real time (SMRT) sequencing is a third-generation sequencing technology which offers great improvement than NGS on reads length and avoids the requirement of assembly in NGS^6,7^. The combination of SMRT and NGS has proceeded the genome assembly and transcriptomic research in several species. The genome assembly of (sunflower) *Helianthus annuus* and an *indica* rice Shuhui498 (R498) was completed with PacBio SMRT technology^8,9^. The combination of these 2 sequencing technologies has also been applied in many ways, such as seeking for the characteristic of transcriptomes and identifying new genes in *Sorghum bicolor*^10^, *Zea mays*^11^, *Phyllostachys edulis*^12^. For species without genome information published, the combination of NGS and SMRT was applied to establish a reliable training set for gene prediction and settle biological questions in *Beta vulgaris*^13^, *Alternanthera philoxeroides*^14^, and *Cassia obtusifolia*^15^. In consideration of the absence of tree peony genome, the information of completed mRNA of transcripts is still unclear, which further limits the exploration of tree peony. Therefore, it is necessary to conduct a combined transcript sequencing for the gene prediction and the floral development research in tree peony.

In this study, we performed both SMRT and NGS to generate large-scale full-length transcripts and collect the gene expression profile for bud development of tree peony. Additionally, the data quality was assessed to verify their reliability. The full-length transcripts will provide gene sequence information for the further study of tree peony, and the gene expression profile will provide comprehensive understanding of the bud development of tree peony.

## Methods

### Design and sample collection

**Fig. 1:**
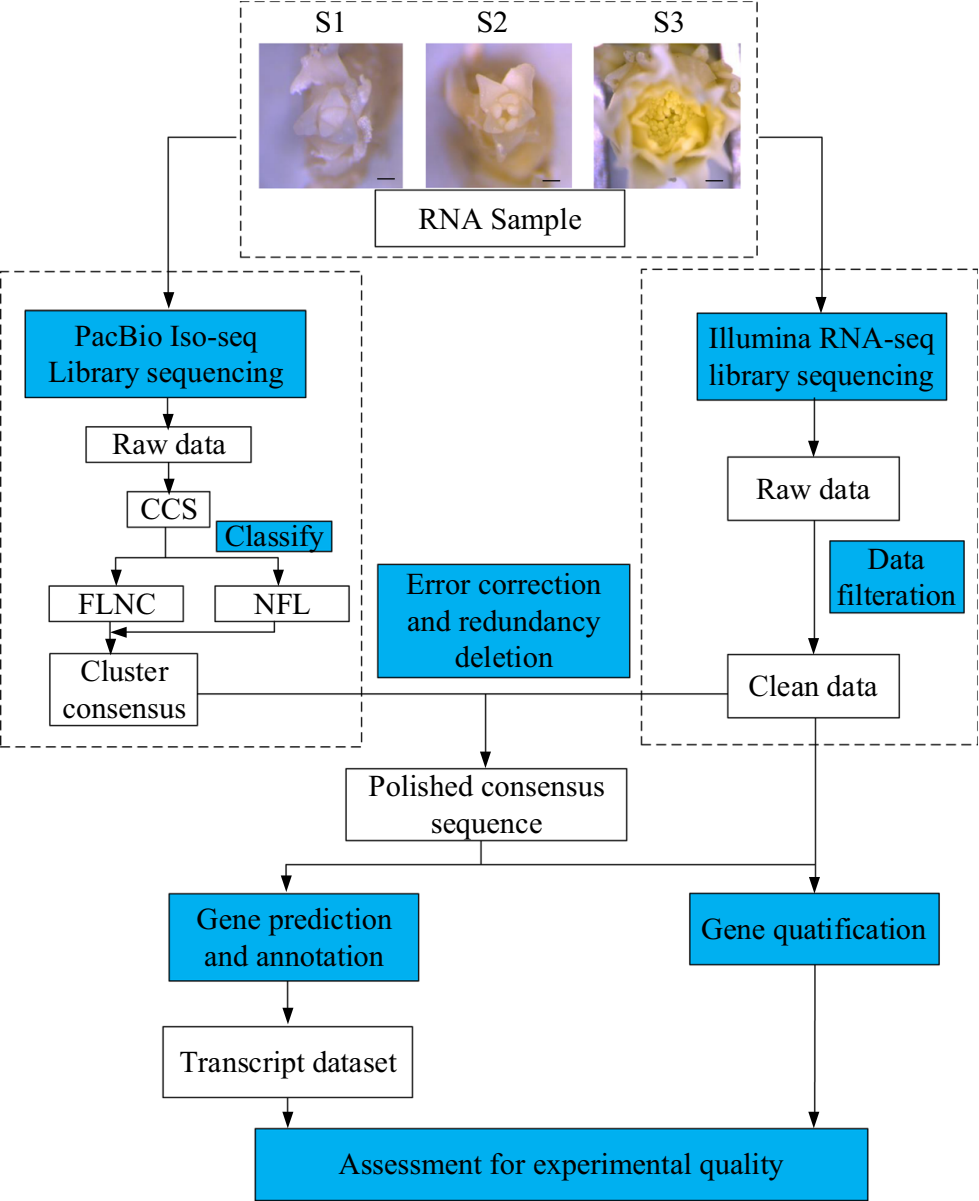
Overview of the experimental design and analysis pipeline. The RNA of ‘High Noon’ bud in its three developmental stages of was collected for PacBio Iso-sequencing and Illumina RNA-seq. The raw data of PacBio Iso-seq were classified and corrected to generate the high-quality transcript sequence. Then these sequences were used to predict the gene sequence and annotate the gene function. The raw data of Illumina RNA-seq were filtered using ng_QC package. Then the clean data were mapped to the full-length transcript sequences by RSEM and used to calculate the reads coverage and gene abundance with package DESeq R package and DESeq2, respectively. The sample stage was confirmed through the parallel samples observed through microscope. Stage I represents the vegetative stage. The stage II represents the bud developmental stage during which the floral organ differentiated. Stage III represents the stage during which the floral organ were formed and the floral bud was identified. Bar = 0.5 mm.

‘High Noon’ is a cultivar of tree peony, which contributed an important genetic resource for extending flowering period. The buds of different developmental stage were obtained from 3–5 years-old plants of a farm in Heze (E, 115°32′30.7818″; N, 35°20′4.794″). After discarding the adjacent scales and leaves, the buds were transferred to liquid nitrogen immediately and stored to −80 °C. The buds were also fixed simultaneously in FAA solution as parallel samples for microscopic observation. And subjected to section in slices and observed under microscope (Zeiss Primo Star, Germany). Through paraffin sections, vegetative meristem (Stage I, S1), floral meristem (Stage II, S2) and floral organ (Stage III, S3) were identified each with at least 3 buds (Fig. 1).

### RNA extraction, Pacbio cDNA library preparation and sequencing

Total RNA was extracted using RNeasy Plant Mini kit (Qiangen, 74904) and treated with RNase-free DNase I (TAKARA, D2215) according to the manufacture’s protocol. The RNA was used for cDNA synthesis through SMARTer PCR cDNA Synthesis Kit (Clontech). The first strand and second strand were synthesized with SMARTScribe RT, using oligo(dT) primer and PCR Primer, respectively. Then the cDNA was selected with the BluePippin Size Selection System (Sage Science, Beverly, MA) according to the Isoform Sequencing protocol as described by Pacific Biosciences (PN 101-070-200-02). To increase the sequencing yield of >4 kb transcripts, a mixture of unfiltered fractions and fractions with size of >4 kb with a mole ratio of 1:1 was processed with the DNA Template Prep Kit (Pacific Biosciences of California, Inc.). Then the library was ready for sequencing after a binding of primer and DNA polymerase to the mixed transcripts. The final library was sequenced on Pacific Bioscience RS II platform (Pacific Biosciences of California, Inc.) by Novogene technology (Tianjin, China; http://www.novogene.com/).

### Illumina cDNA library construction and sequencing

After total RNA was extracted as above, mRNA was enriched by Oligo dT beads and broke into short fragment in fragmentation buffer. Then the first-strand cDNA and second-strand cDNA was synthesized using random hexamers and dNTPs, respectively. The cDNA was subjected to purification and size fractioned by AMPure XP beads, with end pairing, “A” base and Illumina adapter ligation. Then the cDNA libraries were generated by a PCR amplification. After quality control with an Agilent2100 Bioanalyzer, the cDNA libraries were sequenced with a PE mode of 150 bp on an Illumina HiSeq 2000 platform by Novogene technology (Tianjin, China; http://www.novogene.com/).

### Data filtering and error correction

Sequence data were processed using the SMRTlink 5.1 software. Circular consensus sequence (CCS) was generated from the raw subreads with a parameter of minimum length > 200 and minimum predicted accuracy > 0.8. The generated CCS sequences were then classified into Full-length non-chimeric reads (FLNC) and non-full length non-chimeric reads (NFL) according to the containment of 5′ primer, 3′ primer and poly A. FLNC were then fed into the cluster step, which underwent an isoform-level clustering (ICE), followed by a final Arrow polishing with NFL, with a minimum accuracy of 0.99. The resulting consensus reads were subjected to a correction using the Illumina RNA-seq data with the software LoRDEC. Then, after a redundancy deletion by CD-HIT software (−c 0.95, −aS 0.99), the final high quality, full-length, polished consensus sequences were generated after a redundancy deletion by CD-HIT software.

### Gene quantification

The raw reads of Illumina RNA-seq were filtered by software ng_QC (−t 4, −L 20, −N 0.001). The clean data was mapped to the Polished consensus sequence by bowtie2 using end-to-end and sensitive mode. The readcounts of each transcript were calculated using RSEM and transformed into FPKM value. The expressional differential analysis was conducted by DESeq R package with a criterion of fold change > 2 and qvalue < 0.001.

## Data Records

The sequencing raw data and files of gene abundance analysis in this study were deposited in NCBI Gene Expression Omnibus (GEO) and NCBI Sequence Read Archive (SRA) with accessions GSE133476 and SRP212254^16,17^. The annotation information of full-length transcripts in this study was deposited in figshare^18^. The Supplementary material including quality assessment data of raw reads was deposited in figshare^18^. The differentially expressed gene list relative to plant hormone biosynthesis and signaling pathways was deposited in figshare^18^. The flow cytometry analysis of ‘High Noon’ was deposited in figshare^18^.

## Technical Validation

### RNA qualities

The purity and integrity of the total RNA was assessed with Nanodrop 2000 and Agilent 2100. The RNA samples with RIN > 8.0 were used for sequencing library. Qubit 2.0 was used to measure the quantity of RNA sample and cDNA library. The RNA quality values in this study are listed in Table 1.

**Table 1:** Summary of sequencing strategies in this study.

Samples	Sequencing Strategy	Library Layout	Platform	Instrument model	Tissue	OD260/280	OD260/230	25S/18S	RIN
S1-1	RNA-seq	paired	Illumina	Illumina HiSeq 2000	floral bud	2.194	1.388	2.1	8.8
S1-2	RNA-seq	paired	Illumina	Illumina HiSeq 2000	floral bud	2.326	0.877	1.4	8.3
S1-3	RNA-seq	paired	Illumina	Illumina HiSeq 2000	floral bud	2	1.053	1.6	9.1
S2-1	RNA-seq	paired	Illumina	Illumina HiSeq 2000	floral bud	2.115	1.743	1.8	10
S2-2	RNA-seq	paired	Illumina	Illumina HiSeq 2000	floral bud	2.069	2.011	1.8	9.3
S2-3	RNA-seq	paired	Illumina	Illumina HiSeq 2000	floral bud	2.152	2.073	1.9	9.8
S3-1	RNA-seq	paired	Illumina	Illumina HiSeq 2000	floral bud	2.161	1.463	1.8	9.6
S3-2	RNA-seq	paired	Illumina	Illumina HiSeq 2000	floral bud	2.153	1.859	2	10
S3-3	RNA-seq	paired	Illumina	Illumina HiSeq 2000	floral bud	2.092	0.823	1.8	9.3
HN	RNA-seq	single	PACBIO_SMART	PacBio RS II	floral bud	mixed	mixed	mixed	mixed

### Pacbio ISO-seq quality validation

A total of 15.94 Gb raw data was generated by 15,654,254 subreads in the Pacbio ISO-seq. After a single molecular self-correction, circular consensus sequences (CCSs) of 714,643 reads was obtained, which was subsequently classified to full-length non-chimeric (FLNC) with 5′ primer, 3′ primer and poly A and non-full length (NFL) with a proportion of 61.78% and 38.22%, respectively. Consequently, a total of 441,507 high-quality FLNC reads was obtained through the cluster of FLNC and correction by NFL.

As SMRT sequencing generates a high error rate, it is necessary to perform error correction, which includes self-correction by iterative clustering of circular-consensus reads and correction with high-quality NGS short reads. To this end, the NGS sequence data in this study was used to correct the SMRT sequences using LoRDEC software. After that, redundant transcripts were removed by CD-HIT, and a total of 115,439 non-redundant transcripts (Polished consensus sequences) with an average length of 2,060 bp were obtained (see Table 2).

**Table 2:** Statistic of ISO-sequencing in this study.

Type	Total_nucleotides (bp)	Total_reads_number	Average length (bp)
subreads	15,936,030,572	15,654,254	1,018
CCS	1,236,332,390	714,643	1,730
FLNC	760,275,054	441,507	1,722
Consensus	397,594,800	214,800	1,851
polished consensus	387,499,200	214,800	1,804
Before_correct	387,469,755	214,800	1,804
After_correct	388,005,257	214,800	1,807
CD-HIT	237,754,844	115,439	2,060

### Predictions of coding sequence (CDS) and function annotation

**Fig. 2:**
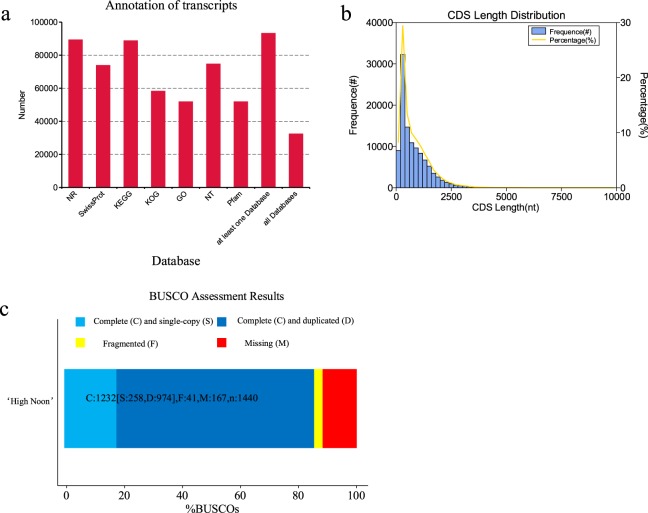
The reads and alignment quality in RNA-seq. (**a**) Per sequence quality scores. The x-axis represents the mean sequence quality of RNA-seq reads. The y-axis represents the count of reads with specified Phred score. (**b**) Per base sequence quality. The x-axis represents the position of reads in RNA-seq. The y-axis represents the Phred scores of each base. (**c**) Per sequence GC content. The x-axis represents the percentage of GC of each read. The y-axis represents the count of the reads with. (**d**) Distribution of insert size after alignment. The x-axis represents the insert size of library after alignment. The y-axis represents the frequency of each size.

To obtain comprehensive information of gene function in tree peony, the 115,439 transcripts were mapped to 7 databases, including NR, NT, Pfam, KOG, Swiss-Prot, KDGG, GO for the gene annotation. As a result, at least 32,416 transcripts could be mapped to all these seven databases (Fig. 2a). The length distribution of successfully annotated genes was showed in Fig. 2b. The completeness of transcripts generated by CD-HIT was assessed by BUSCO 2.3. The results showed that 83.68% transcripts were complete of which single copy BUSCOs and duplicated BUSCOs account for 25.97% and 57.71%, respectively. Of the total 1,440 BUSCO groups searched, only 52 fragmented BUSCOs and 183 missing BUSCOs were found in our database (Fig. 2c). All these results showed that our database was complete and available for subsequent research.

### Illumina RNA-seq quality validation and floral development gene identification

**Fig. 3:**
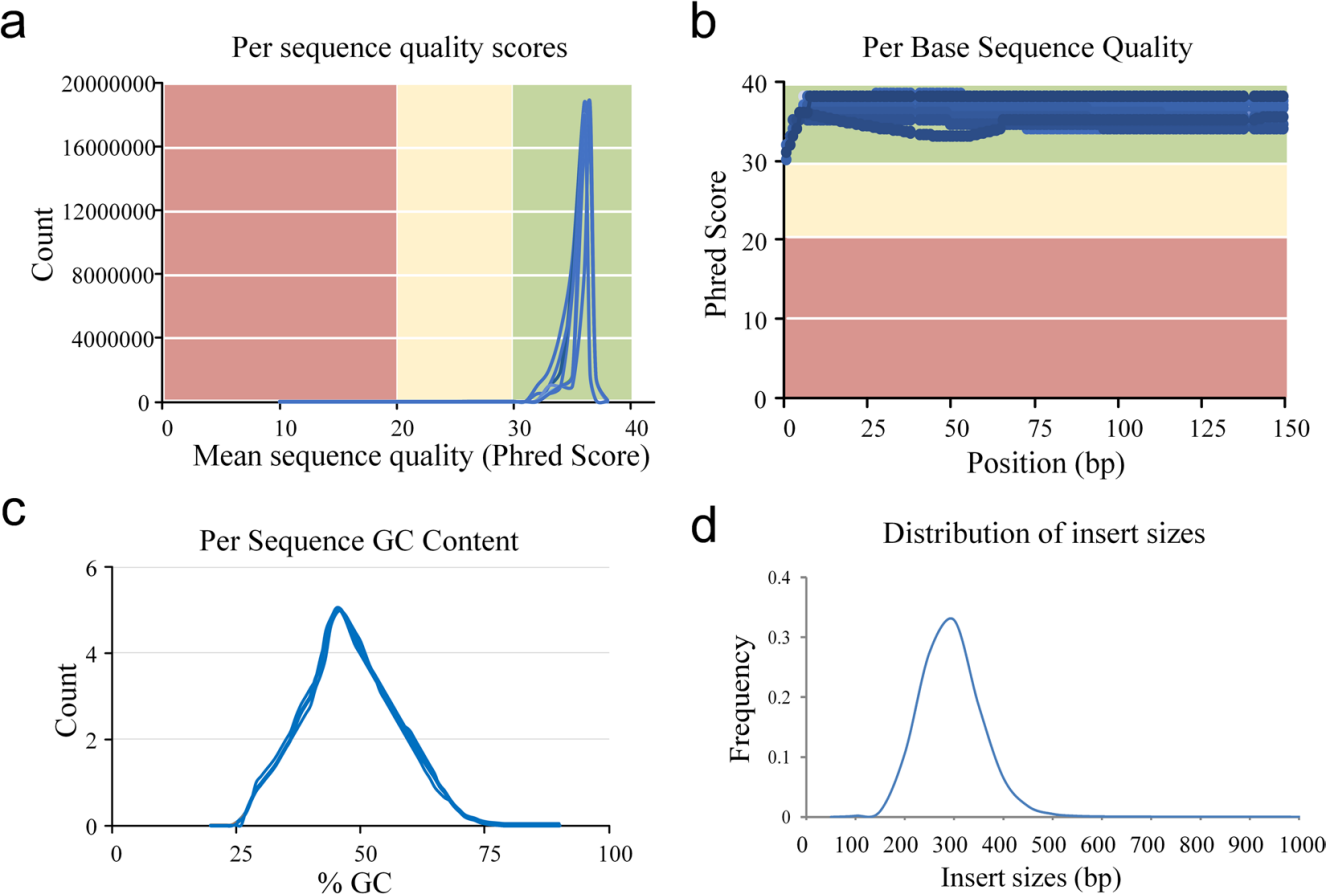
The statistic results of gene annotation and predicted CDS length distribution. (**a**) The transcripts were annotated in 7 databases. The y-axis represents the annotated gene number. (**b**) The CDS length distribution of transcripts in ISO-seq. The x-axis represents CDS length. The y-axis in left represent the frequency of different CDS length. The right y-axis represents the percentage of different CDS length. (**c**) The completeness of transcripts in ISO-seq. The completeness of transcripts was assessed by benchmarking universal single-copy ortholog (BUSCO). The x-axis represents the percentage of detected BUSCOs. The light blue diamond represents the complete (C) and single-copy (S) genes; the dark blue represent complete and duplicated (D) genes; the yellow diamond represents fragmented (F) genes; the red diamond represents the missing (M) genes. Total number of core genes queried was 1440.

**Fig. 4:**
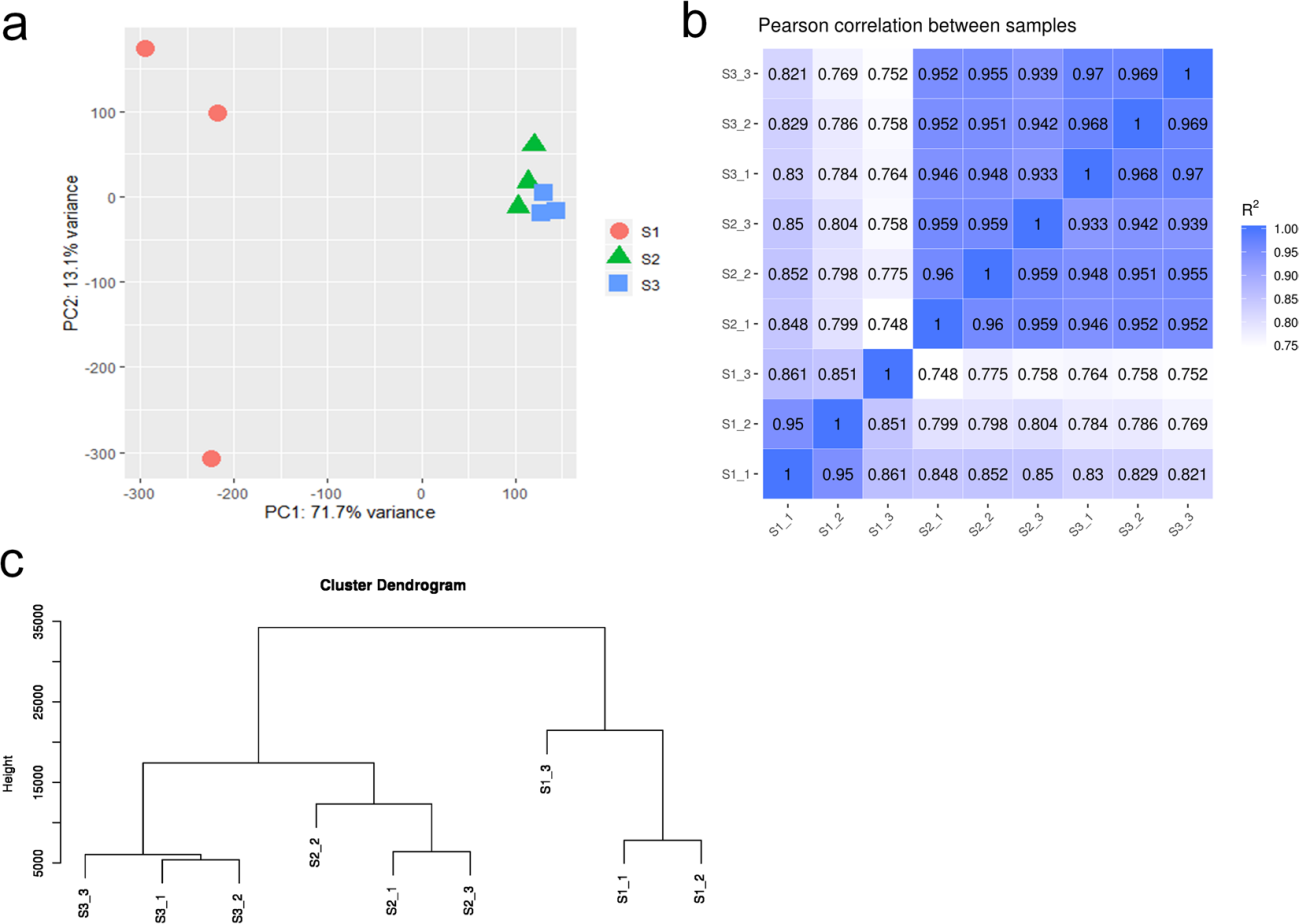
Summary of the sample clustering. (**a**) Principal component analysis of the 9 samples based on expression levels. (**b**) The person correlation between the 9 samples based on expression levels. (**c**) The cluster analysis of the 9 samples based on expression levels.

The reads quality of clean reads in Illumina RNA-seq was assessed using FastQC, including the mean per sequence quality scores, per base quality scores, and GC contents. The per base quality scores were higher than phred quality 30, and most sequences had a quality over 20 (Fig. 3a,b). The GC contents of the samples showed a similar normal distribution, which indicated a sequencing data free of contamination (Fig. 3c). The reads quality of the samples showed that the RNA-seq reads in this study have a high quality. The clean reads of 9 samples were aligned to the 115,439 non-redundant transcripts (reference) using bowtie2 with end-to-end and sensitive mode. The distribution of library insert length after alignment was measured which showed a 270–320 bp distribution (Fig. 3d). The mapping rate of Illumina RNA-seq reads to the high-quality polished sequence ranged from 83.85–88.27% (Table 3). The reliability of the RNA-seq data between the 9 samples was measured with PCA analysis, Pearson correlation and clustering analysis (Fig. 4a–c). The results all showed a reliable biological duplication, indicated that the data obtained in this study could be used for subsequent research.

**Table 3:** Summary of Illumina RNA-sequencing.

Sample	Raw Reads	Clean Reads	Clean Bases (G)	Total mapped reads	Mapping rate (%)
S1_1	49,022,606	48,474,096	7.27	41,483,460	85.58
S1_2	48,375,304	47,857,452	7.18	40,130,002	83.85
S1_3	50,839,914	49,433,588	7.42	41,993,190	84.95
S2_1	58,645,596	58,018,926	8.7	50,391,040	86.85
S2_2	55,158,816	54,515,864	8.18	47,983,332	88.02
S2_3	46,277,122	45,839,116	6.88	40,461,148	88.27
S3_1	47,068,706	45,955,414	6.89	40,193,166	87.46
S3_2	48,714,736	47,383,326	7.11	41,088,574	86.72
S3_3	52,899,086	51,759,904	7.76	45,315,526	87.55

After mapping to the non-redundant transcript, the gene expressional level was analyzed and the differential expressional genes (DEGs) were screened with a parameter of fold change > 2 and q value < 0.001. According to the annotation, DEGs relative to floral development and regulation were analyzed. A total of 143 genes in plant hormone biosynthesis and signaling pathways including auxin and cytokinin which were believed to regulate the floral initiation and bud development were identified^19^. In addition, a total of 26 floral-developmental-relative-DEGs were identified, which might play important roles in floral development process, including the establishment of floral meristem, the specification of flower organ identity and the regulation of floral organogenesis in this study^20,21^. These DEGs were listed in Table 4 and citation 5. These results indicate that our data were valuable for understanding the floral development in tree peony.

**Table 4:** DEGs relative to floral development.

Gene ID	Gene ID in model plant	Gene Symbol	Iidentity (%)	Alignment length (bp)	Pathway
i1_HQ_HNbud_c49771/f2p0/1159	AT1G24260.1	SEP3	54.47	246	floral developmental processes
i1_LQ_HNbud_c231904/f1p0/1102	AT1G24260.1	SEP3	74.49	247	floral developmental processes
i2_HQ_HNbud_c9324/f2p0/2669	AT4G32980.1	ATH1	56.04	323	floral developmental processes
i2_LQ_HNbud_c47156/f1p3/2045	AT3G03090.1	ATVGT1	58.49	477	floral developmental processes
i1_HQ_HNbud_c27914/f7p7/1955	AT3G03090.1	ATVGT1	58.07	477	floral developmental processes
i1_LQ_HNbud_c91035/f1p1/1218	AT5G60910.1	FUL	68.45	206	floral developmental processes
i4_HQ_HNbud_c4367/f6p1/4832	AT5G67100.1	ICU2	60.67	1556	floral developmental processes
i1_LQ_HNbud_c105452/f1p0/1482	AT5G61850.1	LFY	65.64	390	floral developmental processes
i1_LQ_HNbud_c42466/f1p1/1326	AT1G69490.1	NAP	63.58	162	floral developmental processes
i3_LQ_HNbud_c3353/f1p4/3804	AT4G04885.1	PCFS4	60.67	239	floral developmental processes
i3_LQ_HNbud_c2041/f1p5/3526	AT4G04890.1	PDF2	74.9	741	floral developmental processes
i0_HQ_HNbud_c9787/f2p4/869	AT5G20240.1	PI	56.13	212	floral developmental processes
i0_LQ_HNbud_c42964/f1p0/955	AT5G20240.1	PI	57.35	211	floral developmental processes
i0_LQ_HNbud_c22315/f1p0/746	AT5G37055.1	SEF	70.91	165	floral developmental processes
i0_LQ_HNbud_c30233/f1p0/911	AT3G02310.1	SEPALLATA2	68.8	250	floral developmental processes
i1_LQ_HNbud_c213752/f1p14/1085	AT1G14400.1	UBC1	94.74	152	floral developmental processes
i1_LQ_HNbud_c10259/f1p0/1559	AT1G30950.1	UFO	62.14	420	floral developmental processes
i1_LQ_HNbud_c105095/f1p0/1458	AT1G30950.1	UFO	56.9	420	floral developmental processes
i0_LQ_HNbud_c79621/f1p2/890	AT1G65480.1	FT	79.29	169	floral integrator
i1_LQ_HNbud_c105115/f1p1/1458	AT5G13790.1	AGL15	59.32	236	floral repressor
i1_LQ_HNbud_c133723/f1p1/1875	AT5G13790.1	AGL15	60.17	236	floral repressor
i1_LQ_HNbud_c49765/f1p3/1453	AT1G68840.1	TEM2	63.32	368	floral repressor
i1_HQ_HNbud_c33701/f2p5/1226	AT2G01290.1	RPI2	75.31	243	flower meristem identity
i1_LQ_HNbud_c51781/f2p3/1522	AT2G01290.1	RPI2	73.68	266	flower meristem identity
i1_LQ_HNbud_c128329/f1p0/1130	AT1G69120.1	AP1	69.14	256	flower meristem identity
i1_LQ_HNbud_c51214/f1p0/1256	AT1G69120.1	AP1	69.66	178	flower meristem identity

## Data Availability

CD-HIT: http://www.bioinformatics.org/cd-hit/ (version 4.6.6). BUSCO: https://gvolante.riken.jp/index.html (version 2.3).
